# Infectiile digestive cu Salmonella enterica subspecia enterica rezistente la antibiotice – o problema de sanatate publica

**Published:** 2014

**Authors:** Coculescu Bogdan Ioan, Purcarea Victor Lorin, Pahontu Elena

**Affiliations:** * Universitatea de Medicina si Farmacie „Carol Davila”, Bucuresti, Romania

**Rezumat**

Prezenta lucrare este continuarea unei fractiuni dintr-o tema de doctorat axata în principal pe caracterizarea moleculară a pattern-urilor de antibiorezistenţă constitutivă şi dobândită la tulpini de *Salmonella* producătoare de betalactamaze cu spectru extins (BLSE) si in care s-au urmărit: decelarea sensibilităţii/ rezistenţei la antibiotice şi chimioterapice testate pentru tulpinile luate în lucru, prezenţa/absenţa multirezistenţei la antibiotice a tulpinilor analizate, identificarea şi caracterizarea moleculară cu ajutorul metodei PCR a structurilor genetice codante pentru β-lactamaze existente la nivelul tulpinilor de ***Salmonella enterica*** serovar Typhimurium izolate din focare digestive şi rezistente la antibiotice si supravegherea de laborator a cazurilor de rezistenţă la *Salmonella*.

Utilizarea adecvată a terapiei antimicrobiene în caz de rezistenţă la antibiotice a fiecărei tulpini patogene enterice izolate şi corelarea acesteia cu evoluţia bolii diareice acute, respectiv a toxiinfecţiilor alimentare, este obligatorie. Sindromul diareic infecţios are multiple etiologii, una dintre acestea (*Salmonella* spp.) fiind investigată şi evidenţiată în lucrarea de faţă datorită utilizării mijloacelor adecvate de analiză şi al unui algoritm de abordare complexă a diagnosticului.

Infecţiile digestive determinate de bacili gram negativi producători de ESBL ridică probleme deosebite în practica medicală prin scăderea sensibilităţii la antibiotice datorită achiziţionării unor modalităţi variate de realizare a rezistenţei, printre care şi producerea de ESBL.

Importanţa studiului şi supravegherii sindromului diareic infecţios este reliefată de altfel la nivel mondial, devenind o prioritate a tuturor ţărilor lumii.

Organizaţia Mondială a Sănătăţii (OMS) şi Comisia Europeană (DG SANCO) susţin în acest sens o serie de programe ca: WHO-NET, respectiv, Global Salm Surv (Global *Salmonella* Surveillance) în paralel cu cele 2 sisteme europene de supraveghere în domeniu: ECDC (European Centre for Disease Prevention and Control) fost EARSS (European Antimicrobial Resistance Surveillance System - Sistemul European de Supraveghere a Rezistenţei la Antibiotice) şi ESAC (European Surveillance of Antimicrobial Consumption is a European - Sistemul European de Supraveghere a Consumului la Antibiotice).

La fel de importantă este şi asigurarea calităţii investigaţiilor de laborator, o strategie importantă a Programelor Europene de Control Extern (de exemplu, UK NEQAS - United Kingdom National External Quality Assessment Service), posibilă prin participarea laboratoarelor clinice în sistemul de supraveghere/monitorizare a rezistenţei la antibiotice (EARSS).

**Cuvinte-cheie:** infectii digestive, Salmonella enterica, antibiotice

